# Supplementary material for: Activation of Cdc42 GTPase upon CRY2-Induced Cortical Recruitment Is Antagonized by GAPs in Fission Yeast
Source: Cells. 2020 Sep 12;9(9):2089. doi: 10.3390/cells9092089 (PMC7565336; doi:10.3390/cells9092089)
Supplement: Supplementary file 1 [file cells-09-02089-s001.pdf]

Article

Activation of Cdc42 GTPase upon CRY2-Induced Cortical Recruitment is Antagonized by GAPs in Fission Yeast

Iker Lamas, Nathalie Weber and Sophie G. Martin *

Department of Fundamental Microbiology, Faculty of Biology and Medicine, University of Lausanne, Biophore building, 1015 Lausanne, Switzerland; iker.lamasherrera@unil.ch (I.L.); nadliweber@hotmail.com (N.W.)

* Correspondence: Sophie.Martin@unil.ch

Received: date; Accepted: date; Published: date

Figure S1

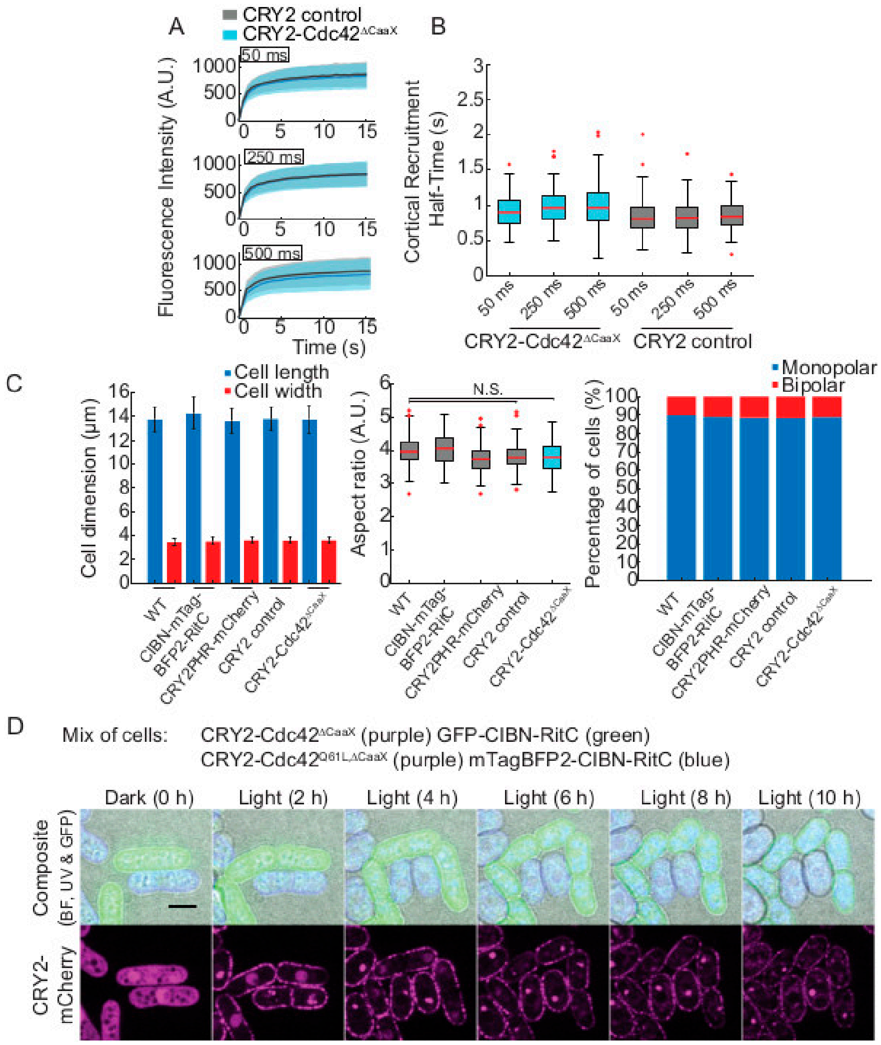

Figure S1. Controls for CRY2-Cdc42$^{\Delta CaaX}$ optogenetic recruitment. (**A**) Plasma membrane recruitment dynamics of CRY2 and CRY2-Cdc42$^{\Delta CaaX}$ in response to periodic 50 ms (top), 250 ms (middle) and 500

ms (bottom) blue-light (λ = 485 nm) pulses (N = 3; n = 30 cells per experiment). Thick line = average; shaded area = standard deviation. CRY2 values are from (Lamas et al., 2020). (**B**) Plasma-membrane recruitment half-times for CRY2 and CRY2-Cdc42$^{\Delta CaaX}$. On each box, the central mark indicates the median; the bottom and the top edges indicate the 25th and 75th percentiles, respectively; the whiskers extend to the most extreme data points not considering outliers, which are plotted individually using the red “+” symbol. (**C**) Cell length and width measurements, aspect ratio and bipolarity of calcofluor-stained cells growing in the dark. CRY2 and CRY2-Cdc42$^{\Delta CaaX}$ do not have any effect on cell morphology ($p^{WTvsCRY2}$ = 0.19; $p^{WTvsCRY2\text{-}Cdc42}$ = 0.19; $p^{CRY2vsCRY2\text{-}Cdc42}$ = 0.83). Monopolarity and bipolarity were assessed on septating cells. (**D**) Blue light–dependent induction of isotropic growth in CRY2-Cdc42$^{Q61L\Delta CaaX}$ (blue cells), but not CRY2-Cdc42$^{\Delta CaaX}$ (green cells) cells photoactivated at 10-min interval (GFP, RFP, and BF channels were acquired every 10 min; UV channel every 1 h). Note that the patchy appearance of CRY2 is likely due to the clustering properties of this protein. Bars = 5 µm.

Figure S2

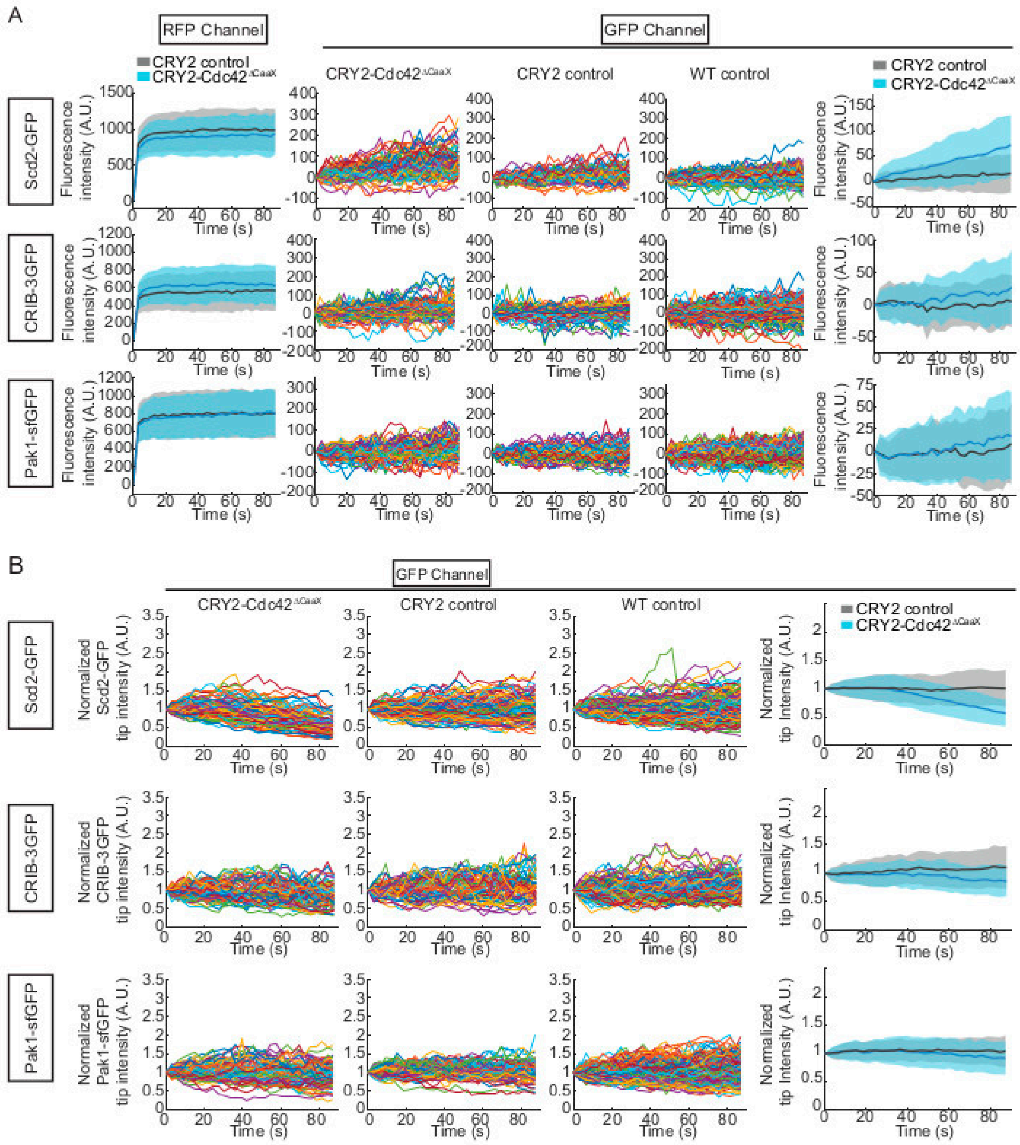

Figure S2. Control and single-cell traces for optogenetic recruitment in WT cells. (**A**) Single-cell traces corresponding to the average plots shown in Fig 1C-E-G. The left column shows the average RFP signal at the plasma membrane of wildtype CRY2-Cdc42$^{\Delta CaaX}$ and CRY2 cells. The 4 other graphs show, from left to right, single cell GFP traces of CRY2-Cdc42$^{\Delta CaaX}$, CRY2, WT control cells and average traces of CRY2-Cdc42$^{\Delta CaaX}$ and CRY2 cells for Scd2-GFP, CRIB-3GFP and Pak1-sfGFP in otherwise wildtype cells. The average traces are identical to Fig 1C-E-G but with standard deviation, showing the biological variability. (**B**) Single-cell traces corresponding to the average plots shown in Fig 1D-F-H. The left 3 columns show, from left to right, single cell tip GFP traces of CRY2-Cdc42$^{\Delta CaaX}$, CRY2 and WT control cells for Scd2-GFP, CRIB-3GFP and Pak1-sfGFP in otherwise WT cells. The right plots show the average cell tip GFP traces of CRY2-Cdc42$^{\Delta CaaX}$ and CRY2 cells for Scd2-GFP, CRIB-3GFP and Pak1-sfGFP in otherwise WT cells (same plots as in Fig 1D-F-H but with standard deviation, showing the biological variability).

Figure S3

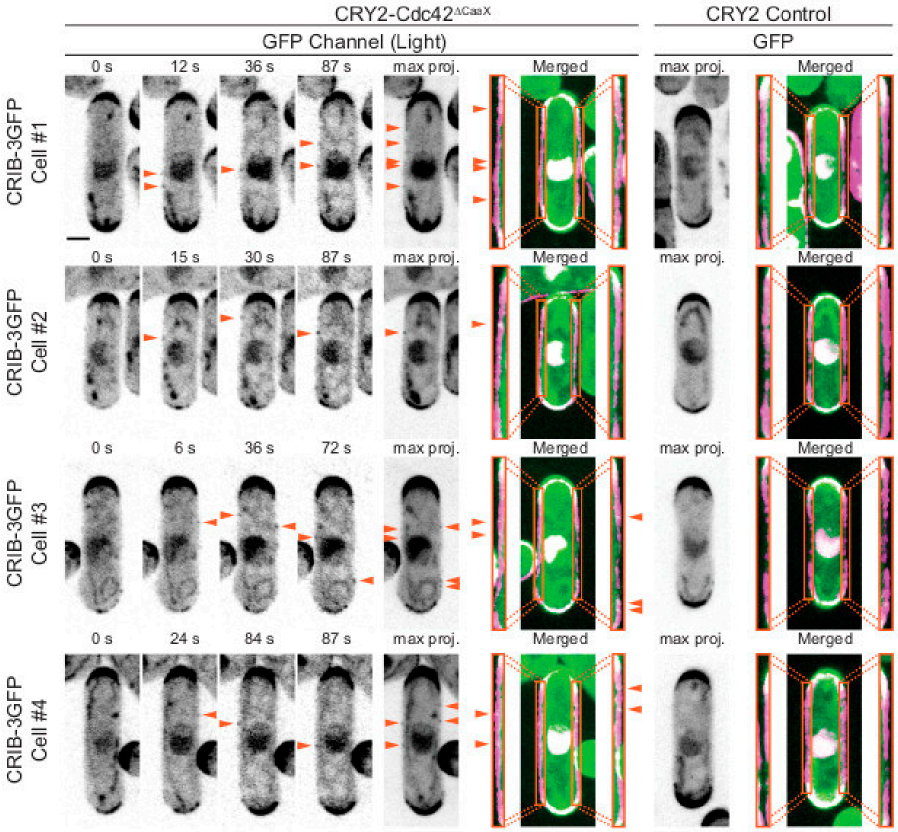

Figure S3. Additional examples for CRY2-Cdc42$^{\Delta CaaX}$ induced CRIB-3GFP cell side recruitment. Localization of CRIB-3GFP in CRY2-Cdc42$^{\Delta CaaX}$-expressing WT cells (B/W inverted images and green channel in merge). The GFP max projection ("max proj.") images show GFP maximum-intensity projections of 30 time points over 87 s. Merged images are composites of GFP and RFP max

projections (t0 omitted from the RFP projection). Magnification of the lateral cortex is shown in the orange insets. Arrowheads point to lateral CRIB-3GFP signal. Autofluorescent organelles appear as linear and circular structures in some of the GFP channel images. Bars = 2 μm.

Figure S4

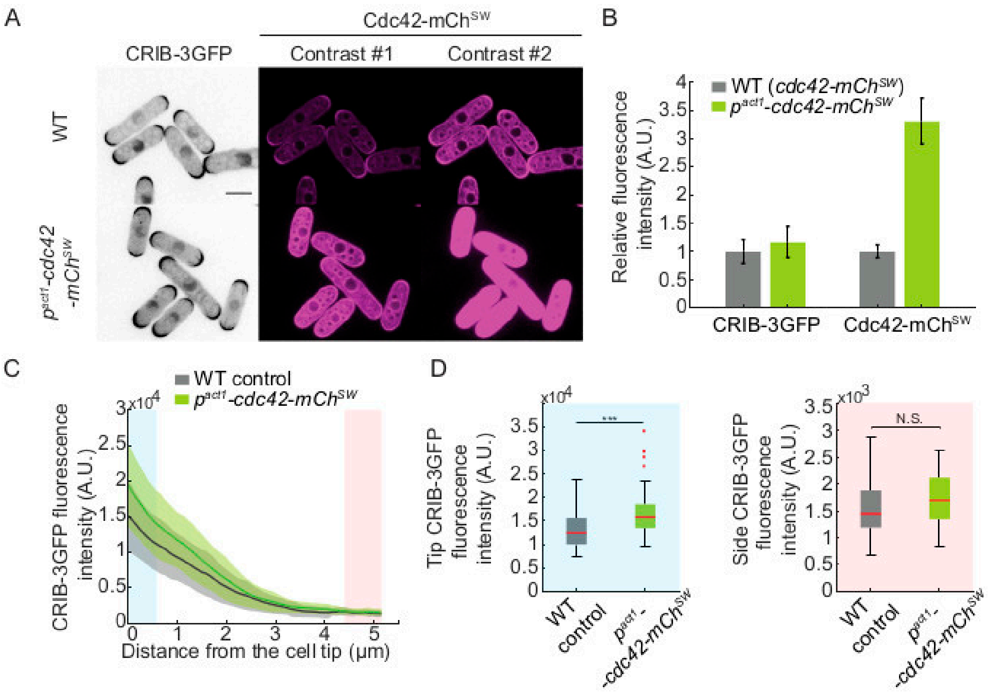

Figure S4. Overexpression of Cdc42-mCh^{SW} in WT cells does not induce ectopic activation of Cdc42. (**A**) *p^{act1}* promoter induces overexpression of Cdc42-mCh^{SW} in CRIB-3GFP cells. Two different contrasting settings are shown. (**B**) Relative expression of CRIB-3GFP and Cdc42-mCh^{SW} in WT (*cdc42-mCherry^{SW}*) and *p^{act1}-cdc42-mCherry^{SW}* cells. *p^{act1}-cdc42* cells exhibit 1.2x and 3.3x higher CRIB-3GFP and Cdc42-mCh^{SW} fluorescence intensities respectively as compared to WT cells. *N* = 1; *n* = 50 cells. (C) Distribution profile of CRIB-3GFP in WT and *p^{act1}-cdc42* cells at the cortex from the centre of cell tips towards the cell side. Blue area represents the centre of the tip (0.5 μm) and red area represents the cell side. The CRIB profile was corrected by the 1.2-fold increase in CRIB expression. (D) CRIB-3GFP fluorescence intensities at the cell tip (left) and cell sides (right) in WT (*cdc42-mCherry^{SW}*) and *p^{act1}-cdc42-mCherry^{SW}* cells derived from the blue and red areas shown in C. $p^{CRIB\text{-}3GFP\text{-}Tip}$ = 3.7e-5; $p^{CRIB\text{-}3GFP\text{-}Side}$ = 0.18. *N* = 1; *n* = 60 half tips. In all graphs, thick line = average; bars & shaded area = standard deviation; WT, wild type; A.U., arbitrary units. Bars = 5 μm.

Figure S5

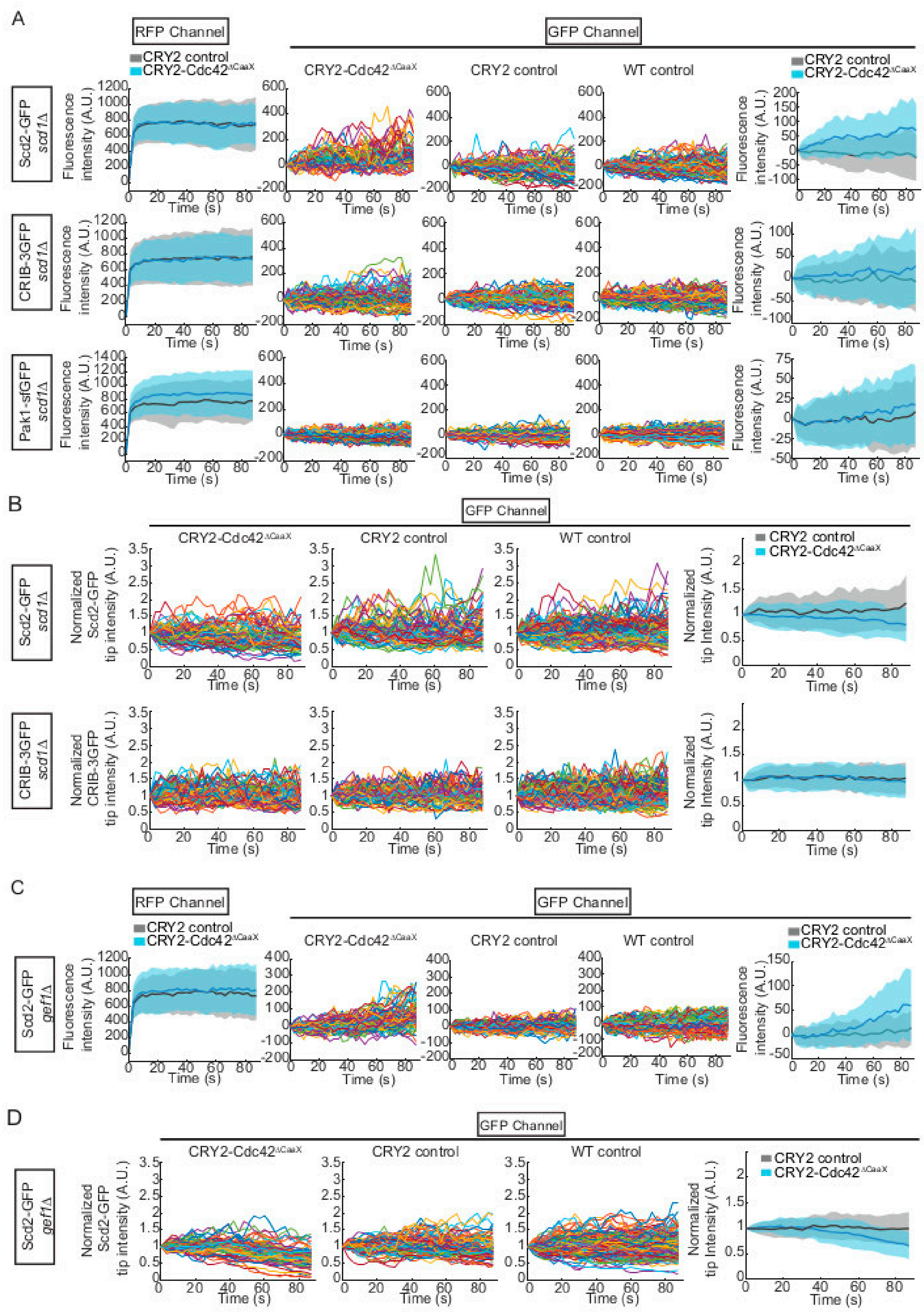

Figure S5. Control and single-cell traces for optogenetic recruitment in *scd1*Δ and *gef1*Δ cells.

(**A**) Single-cell traces corresponding to the average plots shown in Fig 2B. The left column shows the average RFP signal at the plasma membrane of CRY2-Cdc42$^{\Delta CaaX}$ and CRY2 cells. The 4 other graphs

show, from left to right, single cell GFP traces of CRY2-Cdc42$^{\Delta CaaX}$, CRY2, *scd1*Δ control cells and average traces of CRY2-Cdc42$^{\Delta CaaX}$ and CRY2 cells for Scd2-GFP, CRIB-3GFP and Pak1-sfGFP in *scd1*Δ mutants. The average traces are identical to Fig 2B but with standard deviation, showing the biological variability. (**B**) Single-cell traces corresponding to the average plot shown in Fig 2C. The left 3 columns show, from left to right, single cell tip GFP traces of CRY2-Cdc42$^{\Delta CaaX}$, CRY2 and *scd1*Δ control cells for Scd2-GFP and CRIB-3GFP in *scd1*Δ mutants. The right plots show the average cell tip GFP traces of CRY2-Cdc42$^{\Delta CaaX}$ and CRY2 cells for Scd2-GFP and CRIB-3GFP in *scd1*Δ cells (same plots as in Fig 2C but with standard deviation, showing the biological variability). (**C**) Single-cell traces corresponding to the average plots shown in Fig 2E. The left column shows the average RFP signal at the plasma membrane of wildtype CRY2-Cdc42$^{\Delta CaaX}$ and CRY2 cells. The 4 other graphs show, from left to right, single cell GFP traces of CRY2-Cdc42$^{\Delta CaaX}$, CRY2, *gef1*Δ control cells and the average (same plots as in Fig 2E but with standard deviation, showing the biological variability) traces of CRY2-Cdc42$^{\Delta CaaX}$ and CRY2 cells for Scd2-GFP in *gef1*Δ mutants. The average traces are identical to Fig 2E but with standard deviation, showing the biological variability. (**D**) Single-cell traces corresponding to the average plots shown in Fig 2F. The left 3 columns show, from left to right, single cell tip GFP traces of CRY2-Cdc42$^{\Delta CaaX}$, CRY2 and *gef1*Δ control cells for Scd2-GFP in *scd1*Δ mutants. The right plots show the average cell tip GFP traces of CRY2-Cdc42$^{\Delta CaaX}$ and CRY2 cells for Scd2-GFP in *gef1*Δ cells (same plots as in Figure 2F but with standard deviation, showing the biological variability).

Figure S6

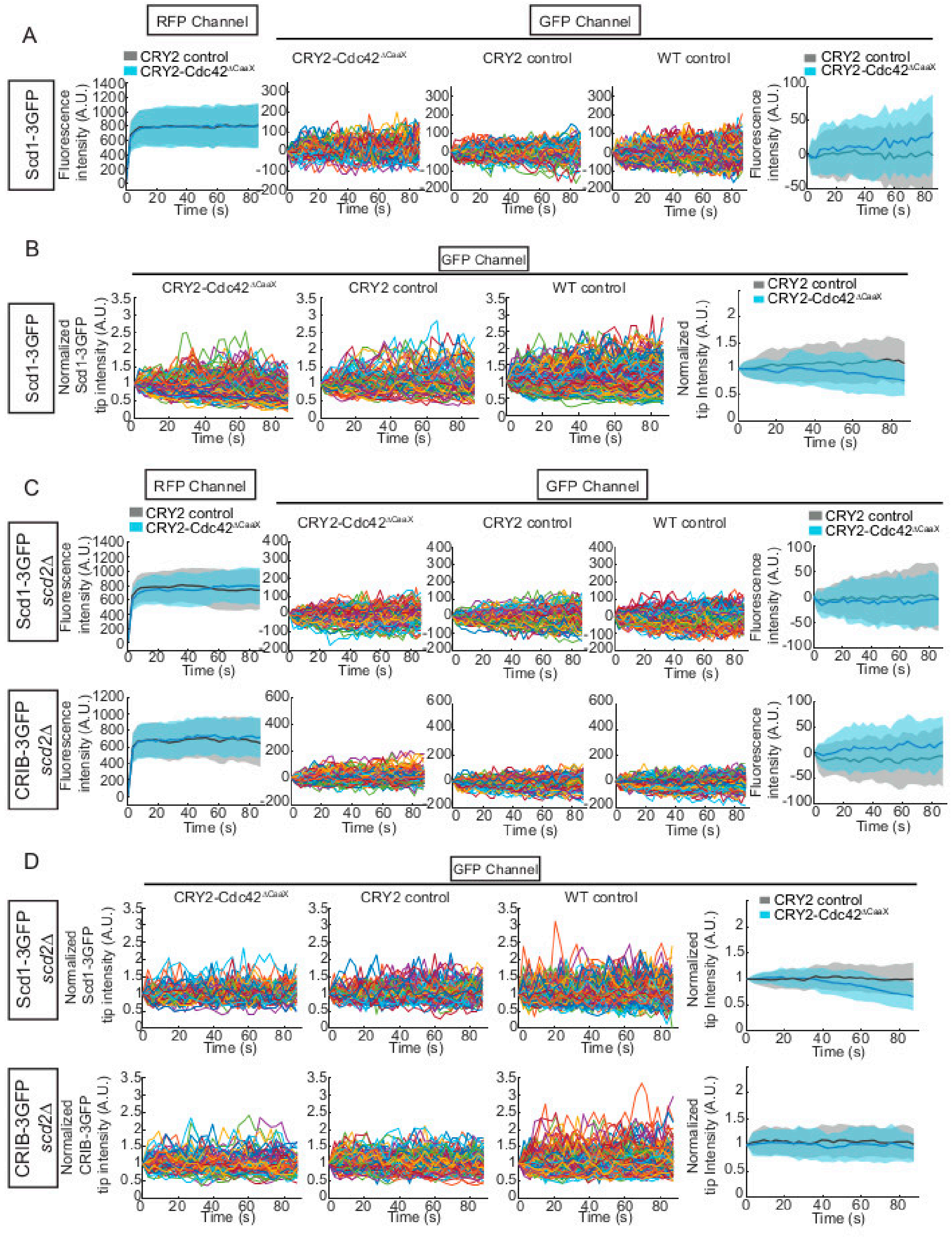

Figure S6. Control and single-cell traces for optogenetic recruitment of Scd1-3GFP and CRIB-3GFP in WT *and sdc2*Δ cells. **(A**) Single-cell traces corresponding to the average plots shown in Fig 3B. The left column shows the average RFP signal at the plasma membrane of wildtype CRY2-Cdc42$^{\Delta CaaX}$ and

CRY2 cells. The 4 other graphs show, from left to right, single cell GFP traces of CRY2-Cdc42$^{\Delta CaaX}$, CRY2, WT control cells and the average traces of CRY2-Cdc42$^{\Delta CaaX}$ and CRY2 cells for Scd1-3GFP in otherwise WT cells. The average traces are identical to Fig 3B but with standard deviation, showing the biological variability. (**B**) Single-cell traces corresponding to the average plot shown in Fig 3C. The left 3 columns show, from left to right, single cell tip GFP traces of CRY2-Cdc42$^{\Delta CaaX}$, CRY2 and WT control cells for Scd1-3GFP in otherwise WT cells. The right plots show the average cell tip GFP traces of CRY2-Cdc42$^{\Delta CaaX}$ and CRY2 cells for Scd1-3GFP in otherwise WT cells (same plots as in Fig 3C but with standard deviation, showing the biological variability). (**C**) Single-cell traces corresponding to the average plots shown in Fig 3E-G. The left column shows the average RFP signal at the plasma membrane of wildtype CRY2-Cdc42$^{\Delta CaaX}$ and CRY2 cells. The 4 other graphs show, from left to right, single cell GFP traces of CRY2-Cdc42$^{\Delta CaaX}$, CRY2, *scd2*Δ control cells and the average traces of CRY2-Cdc42$^{\Delta CaaX}$ and CRY2 cells for Scd1-3GFP and CRIB-3GFP in *scd2*Δ mutants. The average traces are identical to Fig 3E-G but with standard deviation, showing the biological variability. (**D**) Single-cell traces corresponding to the average plot shown in Fig 3F. The left 3 columns show, from left to right, single cell tip GFP traces of CRY2-Cdc42$^{\Delta CaaX}$, CRY2 and *scd2*Δ control cells for Scd1-3GFP in *scd2*Δ mutants. The right plots show the average cell tip GFP traces of CRY2-Cdc42$^{\Delta CaaX}$ and CRY2 cells for Scd1-3GFP in *scd2*Δ cells (same plots as in Fig 3F but with standard deviation, showing the biological variability).

Figure S7

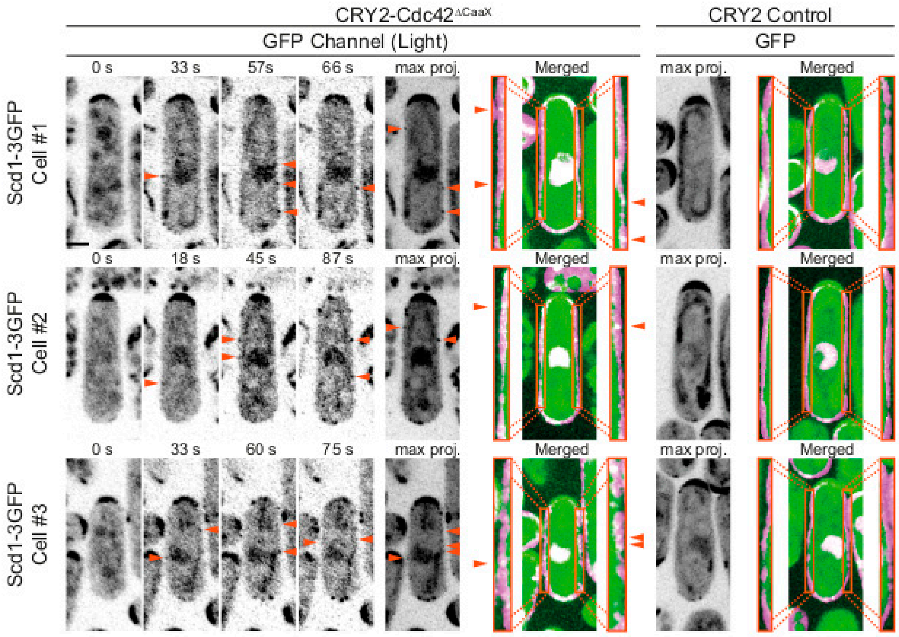

Figure S7. Additional examples for CRY2-Cdc42$^{\Delta CaaX}$ induced Scd1-3GFP cell side recruitment. Localization of Scd1-3GFP in CRY2-Cdc42$^{\Delta CaaX}$-expressing WT cells (B/W inverted images and green

channel in merge). The GFP max projection ("max proj.") images show GFP maximum-intensity projections of 30 time points over 87 s. Merged images are composites of GFP and RFP max projections (t0 omitted from the RFP projection). Magnification of the lateral cortex is shown in the orange insets. Arrowheads point to lateral CRIB-3GFP signal. Autofluorescent organelles appear as linear and circular structures in some of the GFP channel images. Bars = 2 µm.

Figure S8

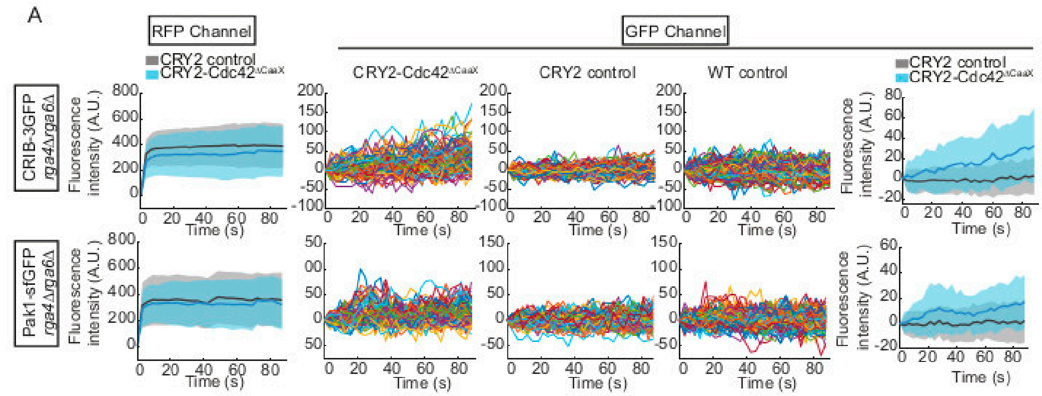

Figure S8. Control and single-cell traces for optogenetic recruitment of CRIB-3GFP and Pak1-sfGFP in rga4Δ*rga6*Δ cells. (**A**) Single-cell traces corresponding to the average plots shown in Fig 5A. The left column shows the average RFP signal at the plasma membrane of wildtype CRY2-Cdc42$^{\Delta CaaX}$ and CRY2 cells. The 4 other graphs show, from left to right, single cell GFP traces of CRY2-Cdc42$^{\Delta CaaX}$, CRY2, *scd2*Δ control cells and the average traces of CRY2-Cdc42$^{\Delta CaaX}$ and CRY2 cells for CRIB-3GFP and Pak1-sfGFP in *rga4*Δ*rga6*Δ mutants. The average traces are identical to Figure 5A but with standard deviation, showing the biological variability.

Table S1: List of strains used in this study.
